# Cachexia Index as a Prognostic Indicator in Patients with Gastric Cancer: A Retrospective Study

**DOI:** 10.3390/cancers14184400

**Published:** 2022-09-10

**Authors:** Can Gong, Qianyi Wan, Rui Zhao, Xinrong Zuo, Yi Chen, Tao Li

**Affiliations:** 1 Department of General Surgery, West China Hospital, Sichuan University, Chengdu 610041, China; 2 Sichuan Cancer Center, Sichuan Cancer Hospital & Institute, School of Medicine, University of Electronic Science and Technology of China, Chengdu 610042, China; 3 Department of Gastrointestinal Surgery, West China Hospital, Sichuan University, Chengdu 610041, China; 4 Department of Anesthesiology, The Affiliated Hospital of Southwest Medical University, Luzhou 646000, China; 5 Laboratory of Mitochondria and Metabolism, Department of Anesthesiology, National Clinical Research Center for Geriatrics, West China Hospital, Sichuan University, Chengdu 610041, China

**Keywords:** gastric cancer, cachexia index, cancer cachexia, overall survival

## Abstract

**Simple Summary:**

Gastric cancer (GC) is one of the most common cancers and fourth for mortality of all malignancies globally. Patients with GC had a high prevalence of cachexia. However, the current diagnostic criteria for cancer cachexia are inconsistent, and the prognostic value of cachexia in GC is controversial. In this study, we investigated the prognostic value of the cachexia index (CXI), a new measurement of cachexia, in 324 patients with GC. We demonstrated that low CXI could be a useful measurement of cachexia, which was verified by analyzing the associations between the CXI and TNM stage, serum nutritional and inflammatory markers, postoperative complications, and overall survival. We also found that the combination of CXI with cachexia, BMI, or TNM stage can more accurately distinguish patients with poor prognoses, which could be helpful to manage and support these patients early.

**Abstract:**

The current diagnostic criteria for cancer cachexia are inconsistent, and the prognostic value of cachexia in gastric cancer (GC) is controversial. This study aimed to investigate the prognostic value of the cachexia index (CXI) in patients with GC. We calculated the CXI as skeletal muscle index (SMI) × serum albumin/neutrophil-lymphocyte ratio (NLR), and a total of 161 and 163 patients were included in the high and low CXI groups, respectively. Low CXI was significantly associated with a more advanced tumor–node–metastasis (TNM) stage, a higher level of serum C-reactive protein, serum interleukin-6, and NLR, but also a decreased level of serum prealbumin and albumin. In addition, patients in the low CXI group were more likely to have postoperative pulmonary infections (9.8% vs. 3.7%, *p* = 0.03). Cox proportional analyses indicated that patients with low CXI (HR 0.45, 95% CI 0.29 to 0.69; *p* < 0.001) or TNM stage III+IV (HR 4.38, 95% CI 2.54 to 7.55; *p* < 0.001) had a significantly poorer overall survival (OS). Kaplan–Meier survival curves suggested that patients with low CXI had a significantly decreased OS, which was not affected by subgroup analyses of different sex, age, cachexia, body mass index (BMI), and TNM stage. Furthermore, low CXI combined with cachexia, low BMI, or TNM stage III+IV caused the worst OS in each subgroup analysis, respectively. Our study demonstrated that CXI had a good prognostic value in GC. Greater attention should be paid to patients with low CXI, particularly those combined with cachexia, low BMI, or TNM stage III+IV.

## 1. Introduction

Gastric cancer (GC) is one of the most common cancers in the gastrointestinal tract. In 2020, there were more than one million new GC cases, making it the fifth most common cancer worldwide [1]. GC is also a highly lethal malignancy; in particular, the median survival for advanced GC is less than 1 year [2]. There were about 769,000 deaths due to GC worldwide in 2020, ranking fourth for mortality of all malignancies globally [1]. Notably, China has nearly half of the new GC cases and deaths every year in the world [1,3], and it is estimated that about 10 million new GC cases will occur in China from 2021 to 2035, leading to about 5.6 million GC deaths [4].

Malignancies, including GC, could cause an increased energy expenditure, excess catabolism, and elevated inflammation of the body, leading to cancer cachexia [5,6]. Cancer cachexia is generally recognized as a multifactorial syndrome, and skeletal muscle mass loss, low nutritional conditions, and elevated inflammation levels are important features of cancer cachexia [6,7]. The clinical diagnostic criteria for cancer cachexia are inconsistent, while weight loss is a common and indispensable criterion [8,9]. It is estimated that the prevalence of cancer cachexia, defined as weight loss over 5% in the previous 6 months, is about 60% in GC, with an average weight loss of over 10% [6]. However, not all patients can accurately remember their body weight, and the risk of recalling bias might increase. Furthermore, advanced GC patients with peritoneal metastasis could have ascites [10], which might mask the actual weight loss. The prognostic value of cancer cachexia defined by weight loss is controversial in GC. Some studies indicated that advanced GC patients with cancer cachexia had significantly worse overall survival (OS) [11,12], while other studies suggested that cachexia could not completely predict OS in GC patients receiving radical surgeries [13,14].

The cachexia index (CXI) is a new index for estimating cachexia and has been reported in recent studies. It is calculated as skeletal muscle index (SMI) × serum albumin/neutrophil-lymphocyte ratio (NLR) [15]. The key clinical features of cancer cachexia are poor nutritional status, systemic inflammation, and reduced skeletal muscle mass. Clinical measures of these features, that is, serum albumin, NLR, and SMI, are independently associated with poor outcomes [16,17,18,19]. All these features are included in the CXI and might therefore be desirable to measure cancer cachexia. Recent studies reported that CXI had significant associations with survival in patients with lung cancer, liver cancer, biliary tract cancer, and aggressive lymphomas [15,20,21,22,23]. However, the prognostic value of CXI in patients with GC remains unclear. The aim of this study is to investigate the associations between CXI and multiple clinicopathological variables and analyze the impact of CXI on the OS of GC patients in China.

## 2. Methods

### 2.1. Patients

Patients diagnosed with GC between July 2016 and September 2021 were retrospectively collected from the Department of Gastrointestinal Surgery of our hospital in this study. The inclusion criteria were: (1) pathology confirmed GC; (2) adult patients; (3) no history of neoadjuvant therapy; (4) the abdominal CT scan was performed in our hospital. On the other hand, the exclusion criteria were: (1) an inability to tolerate radical or palliative surgery; (2) a history of other malignancies. Through the Hospital Information System (HIS) of our hospital, we accessed the medical records, examination reports, and pathological reports. Each patient was routinely followed up after surgery by telephone or at an outpatient clinic, and the newest follow-up data were collected in July 2022. This study was performed based on the Declaration of Helsinki and approved by the ethics committee of West China Hospital.

### 2.2. Assessment of SMI, CXI, and Cancer Cachexia

The original preoperative abdominal CT images of included patients were extracted from our hospital, and Syngo MultiModality Workplace (Siemens Medical Solutions, Forchheim, Germany) was used for analyzing the skeletal muscle area of the third lumbar vertebra (L3) level [24]. The Hounsfield unit (HU) threshold of skeletal muscle was set from −29 to 150 [25,26]. The SMI was calculated as the area of skeletal muscle (cm^2^) of L3/height squared (m^2^) [26,27]. The preoperative blood samples were taken from the anterior cubital vein with fasting of 10 h. NLR was calculated as the number of peripheral neutrophils/the number of peripheral lymphocytes [28], and the level of serum albumin was obtained from the examination reports of the Clinical Laboratory of West China Hospital. The CXI was calculated as SMI (cm^2^/m^2^) × serum albumin (g/dL)/NLR [15]. Cancer cachexia was diagnosed according to the most accepted criteria by Fearon et al.: weight loss over 5% in the past 6 months; weight loss over at least 2% and body mass index (BMI) < 20; or weight loss over at least 2% and sarcopenia [7].

### 2.3. Statistical Analysis

In this study, normally distributed data are presented as mean ± SD, and not normally distributed data are presented as median (inter-quartile range). The *t*-test or Mann–Whitney U test was used for the comparison of continuous data, and the Chi-squared test or Fisher’s exact test was used for categorical data. We used the univariate Cox proportional hazards model for investigating the associations between clinicopathological variables and OS in patients with GC. The variables with a *p*-value of <0.2 in the univariate analysis would be further analyzed with multivariate analysis. Furthermore, Kaplan–Meier survival curves with log-rank tests would be used for analyzing the prognostic value of CXI. The statistical analyses were performed with the use of SPSS version 25.0 and GraphPad Prism version 8.0, and a two-sided *p*-value of <0.05 meant statistical significance in this study.

## 3. Results

**Figure 1:**
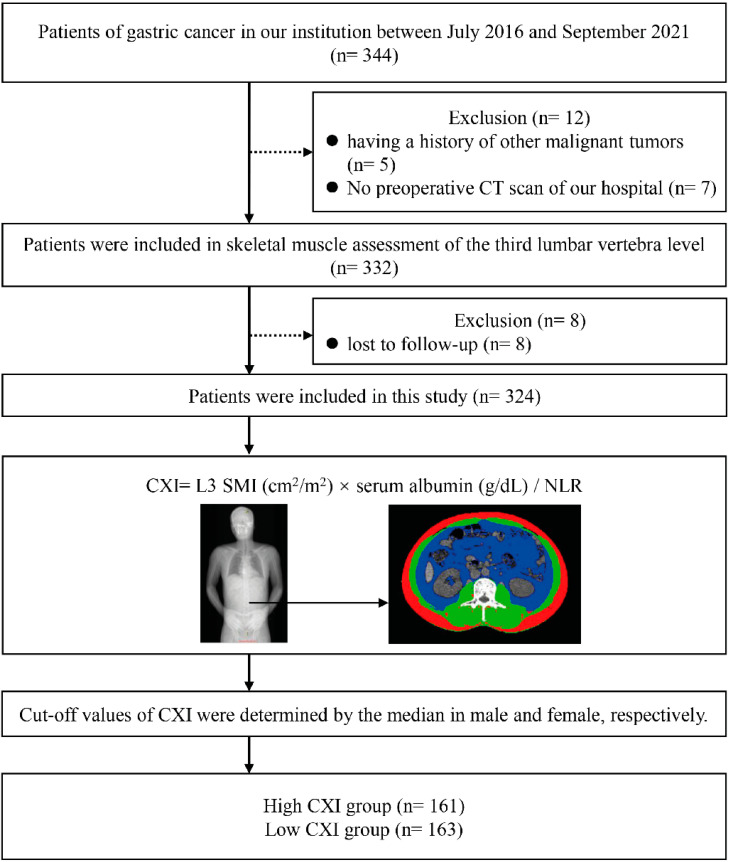
Flow diagram of patients.

We preliminarily identified 344 potentially eligible patients with GC, in which 5 patients had a history of other malignancies and 7 patients had no CT scans. In addition, eight patients were lost to follow-up. Three hundred and twenty-four patients were included in this study, and the CXI was calculated for each patient. Male and female patients were divided into the high and low CXI groups according to their respective median CXI. Overall, 161 patients were included in the high CXI group, and 163 patients were in the low CXI group, respectively (Figure 1).

The mean CXI was 146.20 (±54.24) in the high CXI group and 64.35 (±20.97) in the low CXI group, respectively. There were no significant differences in sex (*p* = 0.97), age (*p* = 0.78), and BMI (*p* = 0.36) between the two groups. Patients in the high CXI group appeared to have a lower rate of cancer cachexia (41.6% vs. 50.9%, *p* = 0.09), but this was not significant (Table 1).

Notably, patients in the low CXI group had a significantly higher rate of advanced tumor–node–metastasis (TNM) stage (*p* < 0.001). No significant differences were found in postoperative adjuvant chemotherapy, smoking, drinking, and comorbidities between the two groups (Table 1). For nutritional and inflammatory markers, a significantly higher level of serum C-reactive protein (CRP) (8.11 ± 15.16 vs. 2.73 ± 4.18 mg/L, *p* < 0.001), serum interleukin-6 (IL-6) (6.03 ± 6.35 vs. 2.91 ± 4.13 pg/mL, *p* < 0.001), and NLR (3.29 ± 1.61 vs. 1.58 ± 0.43 mg/L, *p* < 0.001), but a decreased level of serum prealbumin (194.79 ± 53.21 vs. 227.45 ± 46.55 mg/L, *p* < 0.001) and albumin (3.98 ± 0.45 vs. 4.24 ± 0.37 g/dL, *p* < 0.001), were observed in patients with low CXI (Table 1). Furthermore, patients in the low CXI group were more likely to have postoperative pulmonary infections (9.8% vs. 3.7%, *p* = 0.03), while no significant difference was found in intensive care unit (ICU) admission and abdominal infections between the two groups.

To investigate the impact of multiple clinicopathological variables on the OS of patients, the univariate Cox proportional hazards model was applied. We found that a high BMI (≥22.32) (HR 0.65, 95% CI 0.43 to 0.97; *p* = 0.04) and high CXI (HR 0.65, 95% CI 0.43 to 0.97; *p* < 0.001) were associated with a significantly more favorable OS, respectively. Alternatively, patients with TNM stage III+IV (HR 4.68, 95% CI 2.95 to 7.44; *p* < 0.001) or receiving postoperative adjuvant chemotherapy (HR 2.45, 95% CI 1.19 to 5.06; *p* = 0.02) had a decreased OS (Table 2).

**Figure 2:**
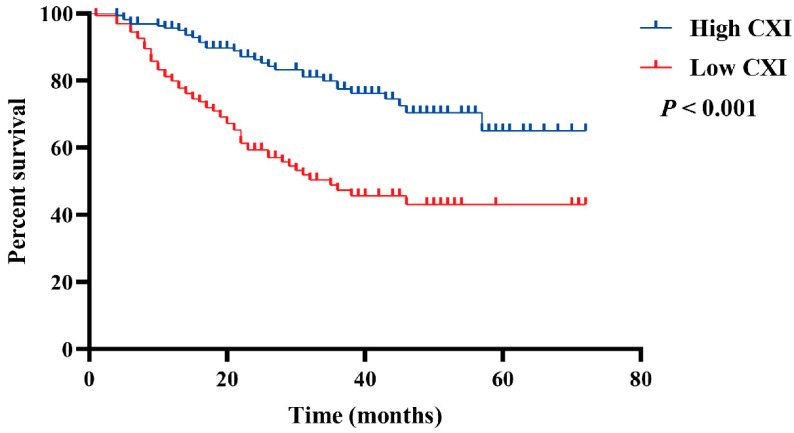
Comparison of OS between patients with low cachexia index and high cachexia index.

In multivariate analysis, only the CXI (HR 0.45, 95% CI 0.29 to 0.69; *p* < 0.001) and TNM stage (HR 4.38, 95% CI 2.54 to 7.55; *p* < 0.001) had significant associations with OS (Table 2). In addition, Kaplan–Meier survival curves with log-rank tests also suggested a significantly more favorable OS in patients with high CXI (*p* < 0.001, Figure 2).

**Figure 3:**
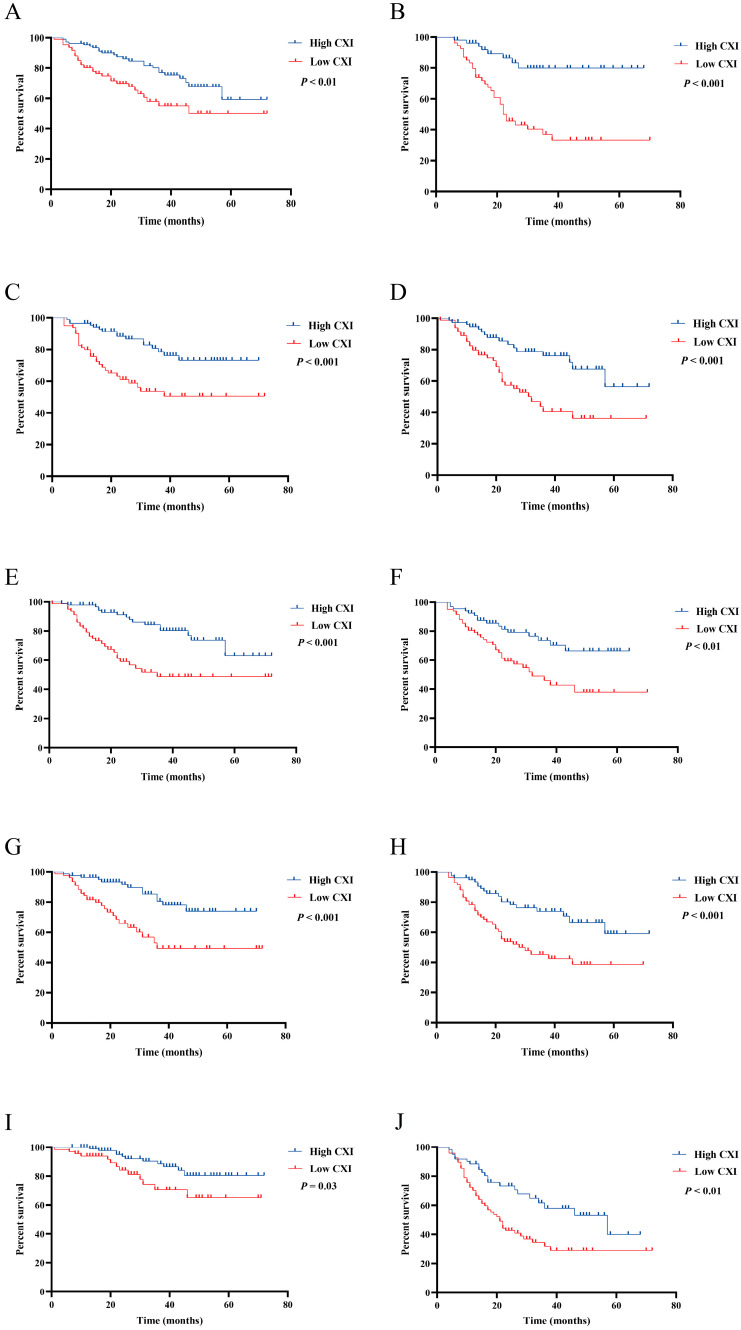
Comparison of OS between patients with low cachexia index and high cachexia index in (**A**) male; (**B**) female; (**C**) patients < 60 years old; (**D**) patients ≥ 60 years old; (**E**) patients without cachexia; (**F**) patients with cachexia; (**G**) patients with BMI ≥ 22.32; (**H**) patients with BMI < 22.32; (**I**) patients with TNM stage I+II; (**J**) patients with stage III+IV.

To further investigate the impact of CXI on OS under different conditions, Kaplan–Meier survival curves with log-rank tests were conducted according to sex, age, cachexia, BMI, and TNM stage. A high CXI was shown to be associated with a significantly more favorable OS in both males and females (Figure 3A,B); patients <60 and ≥60 years old (Figure 3C,D); patients without and with cachexia (Figure 3E,F); patients with BMI ≥ 22.32 and <22.32 (Figure 3G,H); and patients with TNM stage I+II and stage III+IV (Figure 3I,J).

**Figure 4:**
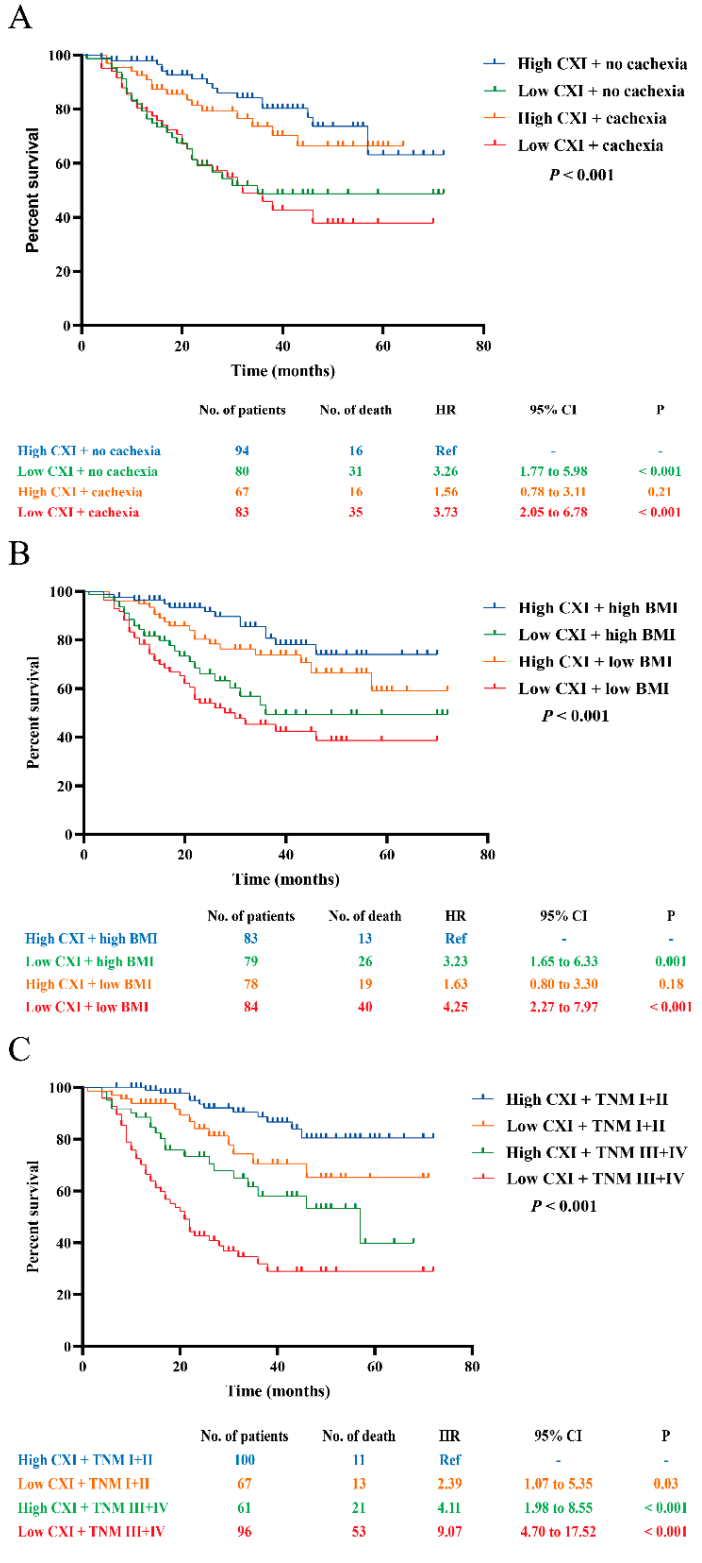
Survival analysis for the combination of CXI and (**A**) cachexia; (**B**) BMI; (**C**) TNM stage.

Moreover, combinations of CXI and cachexia, BMI, and TNM stage were conducted for survival analyses. Compared to those with high CXI + no cachexia, patients with low CXI + cachexia had the worst OS (HR 3.73, 95% CI 2.05 to 6.78; *p* < 0.001), followed by patients with low CXI + no cachexia (HR 3.26, 95% CI 1.77 to 5.98; *p* < 0.001). No significant difference was found between patients with high CXI + no cachexia and high CXI + cachexia (Figure 4A). In combination with CXI and BMI, we found that patients with low CXI + low BMI (<22.32) had the worst OS (HR 4.25, 95% CI 2.27 to 7.97; *p* < 0.001) when compared to those with high CXI + high BMI, followed by patients with low CXI + high BMI (HR 3.23, 95% CI 1.65 to 6.33; *p* = 0.001). There were no significant differences between patients with high CXI + high BMI and high CXI + low BMI (Figure 4B). For the combination of CXI and TNM stage, two of the significant prognostic factors in the multivariate Cox proportional hazards model, we found that patients with low CXI + TNM stage III+IV had the worst OS (HR 9.07, 95% CI 4.70 to 17.52; *p* < 0.001) when compared to those with high CXI + TNM stage I+II, followed by patients with high CXI + TNM stage III+IV (HR 4.11, 95% CI 1.98 to 8.55; *p* < 0.001) and patients with low CXI + TNM stage I+II (HR 2.39, 95% CI 1.07 to 5.35; *p* = 0.03), respectively (Figure 4C).

## 4. Discussion

In this study of 324 GC patients, we calculated the CXI with SMI, serum albumin, and NLR: three cachexia-related parameters. We also observed that patients with low CXI had a significantly higher level of serum CRP and IL-6 but a decreased level of serum prealbumin, indicating that low CXI could be a desirable measurement of cachexia. Although no significance existed, we found that patients in the high CXI group appeared to have a lower rate of cancer cachexia diagnosed by Fearon’s criteria [7], suggesting that low CXI representative cachexia is a little different from that diagnosed by Fearon et al.

Fearon’s criteria are widely applied in diagnosing cancer cachexia, in which weight loss is an indispensable criterion [7]. However, the prognostic value of cachexia diagnosed by Fearon’s criteria is controversial in patients with GC. With Fearon’s criteria, some studies suggested that cancer cachexia was significantly associated with worse OS in advanced GC patients receiving drug treatments [11,12]. However, in GC patients receiving radical surgeries, cachexia was only associated with poor survival at TNM stage II+III instead of stage I [13]. Another prospective study included GC patients of TNM stage I to III and found that preoperative cachexia was only associated with worse OS in young rather than elderly GC patients [14]. Considering that cachexia is associated with the advanced cancer stage [6], we speculated that the cachexia diagnosed by Fearon’s criteria might effectively predict the survival of GC only when patients had marked cachexia. In our study, we found that patients with low CXI had significantly worse OS, and the prognostic value of CXI was not affected by subgroup analyses of different sex, age, cachexia, BMI, and TNM stage. Our study demonstrated that CXI could be a useful prognostic indicator for GC. Furthermore, the multivariate Cox analysis found that only CXI and TNM stages had significant associations with OS, while subgroup analyses about the combination of CXI and TNM stages indicated significant differences in OS. Considering that the TNM stage is a crucial clinicopathological variable and is commonly accepted for guiding postoperative treatment and predicting survival, the combination of CXI and TNM stages could more accurately distinguish patients with different prognoses.

For postoperative outcomes, we found that low CXI was significantly associated with a higher rate of pulmonary infection instead of abdominal infection and ICU admission. We speculated that patients with low CXI had decreased skeletal muscle mass, including respiratory muscle, and the loss of skeletal muscle could lead to an increased risk of postoperative pulmonary complications [29,30]. In addition, hypoproteinemia and elevated inflammation levels were also risk factors for postoperative pulmonary infection [29,31]. Therefore, more attention should be paid to the perioperative management of the respiratory system in patients with relatively low CXI.

Another notable result was that patients with low CXI had a significantly higher NLR and decreased level of serum albumin. Fearon’s criteria mainly included weight loss, BMI, and sarcopenia, while no parameters about nutritional status and systemic inflammation were included. The advantage of CXI over Fearon’s criteria is that it includes NLR and serum albumin. Furthermore, previous studies demonstrated that NLR and serum albumin had significant associations with the prognosis in patients with GC [32,33,34], which might account for why low CXI, instead of cachexia diagnosed by Fearon’s criteria, was significantly associated with worse OS in this study.

There were several strengths in this study. We firstly investigated the prognostic value of CXI in GC, and our sample was the largest among the relevant studies about CXI and the prognosis of other malignancies. In addition, we demonstrated that low CXI could be a useful measurement of cachexia, which was verified by analyzing the associations between CXI and TNM stage, serum nutritional and inflammatory markers, postoperative complications, and OS. We also found that the combination of CXI with cachexia, BMI, or TNM stage can more accurately distinguish patients with poor prognosis, which could be helpful to manage and support these patients early. The limitations of this study were that we determined the low and high CXI by the median CXI of males and females, respectively. There is no unified standard to determine the cut-off value of CXI yet. Our cut-off values may not be suitable for other studies about this issue. Furthermore, this is a single-center and retrospective study, which might increase the risk of selection bias. Our results need more prospective studies to verify them in the future.

## 5. Conclusions

In conclusion, this study demonstrated that CXI could be a desirable measurement of cachexia and have a good prognostic value in GC. Greater attention and early support should be paid to patients with low CXI. Considering the limitations of this study, our results need further studies to verify the results in the future.

## Figures and Tables

**Table 1:** Clinical characteristics between patients in low and high CXI groups.

Characteristics	High CXI (*n* = 161)	Low CXI (*n* = 163)	*p* Value
Male/Female, n	108/53	109/54	0.97
Age, mean ± SD (years)	58.07 ± 11.23	57.69 ± 12.69	0.78
CXI, mean ± SD	146.20 ± 54.24	64.35 ± 20.97	<0.001
BMI, mean ± SD	22.56 ± 3.36	22.23 ± 3.04	0.36
SMI, mean ± SD	50.79 ± 8.60	46.51 ± 8.22	<0.001
Cachexia, *n* (yes/no)	67/94	83/80	0.09
TNM stage, *n*			<0.001
I	55	35	
II	45	32	
III	56	68	
IV	5	28	
Postoperative adjuvant chemotherapy, *n* (yes/no)	132/29	143/20	0.15
Cigarette smoking, *n* (yes/no)	67/94	60/103	0.38
Alcohol drinking, *n* (yes/no)	29/132	39/124	0.19
Hypertension, *n* (yes/no)	27/134	24/139	0.61
Coronary heart disease, n (yes/no)	5/156	3/160	0.46
Diabetes, *n* (yes/no)	13/148	10/153	0.50
Chronic obstructive pulmonary disease, *n* (yes/no)	9/152	10/153	0.84
Serum CRP (mg/L), mean ± SD	2.73 ± 4.18	8.11 ± 15.16	<0.001
Serum IL-6 (pg/mL), mean ± SD	2.91 ± 4.13	6.03 ± 6.35	<0.001
Serum TNF-α (pg/mL), mean ± SD	7.87 ± 5.05	8.11 ± 4.30	0.66
NLR, mean ± SD	1.58 ± 0.43	3.29 ± 1.61	<0.001
Serum PAB (mg/L), mean ± SD	227.45 ± 46.55	194.79 ± 53.21	<0.001
Serum ALB (g/dL), mean ± SD	4.24 ± 0.37	3.98 ± 0.45	<0.001
ICU admission, *n* (yes/no)	10/151	4/159	0.10
Pulmonary infection, *n* (yes/no)	6/155	16/147	0.03
Abdominal infection, *n* (yes/no)	7/154	3/160	0.33

ALB, albumin; BMI, body mass index; COPD, chronic obstructive pulmonary disease; CRP, C-reactive protein; CXI, cachexia index; ICU, intensive care unit; IL-6, interleukin-6; NLR, Neutrophil-lymphocyte ratio; PAB, prealbumin; SD, standard deviation; TNF-α, tumor necrosis factor α; TNM stage, Tumor-node-metastasis stage.

**Table 2:** The Cox univariate and multivariate analysis of overall survival.

Characteristics	Univariate		Multivariate	
	HR (95% CI)	*p*	HR (95% CI)	*p*
Age ≥ 60 years, (<60 as ref)	1.25 (0.84 to 1.86)	0.27		
Male, (female as ref)	0.74 (0.50 to 1.11)	0.15	0.93 (0.62 to 1.41)	0.74
BMI ≥ 22.32 (<22.32 as ref)	0.65 (0.43 to 0.97)	0.04	0.67 (0.44 to 1.04)	0.07
High CXI (low CXI as ref)	0.35 (0.23 to 0.54)	<0.001	0.45 (0.29 to 0.69)	<0.001
Cachexia, (no as ref)	1.36 (0.92 to 2.03)	0.13	0.99 (0.65 to 1.52)	0.99
TNM stage III+IV, (stage I+II as ref)	4.68 (2.95 to 7.44)	<0.001	4.38 (2.54 to 7.55)	<0.001
Postoperative adjuvant chemotherapy, (no as ref)	2.45 (1.19 to 5.06)	0.02	0.80 (0.34 to 1.86)	0.60
Cigarette smoking, (no as ref)	0.98 (0.65 to 1.46)	0.90		
Alcohol drinking, (no as ref)	0.99 (0.61 to 1.62)	0.96		
Hypertension, (no as ref)	1.35 (0.81 to 2.25)	0.26		
Coronary heart disease, (no as ref)	1.38 (0.44 to 4.35)	0.59		
Diabetes, (no as ref)	1.47 (0.74 to 2.92)	0.27		
Chronic obstructive pulmonary disease, (no as ref)	1.21 (0.53 to 2.77)	0.65		

Abbreviations: BMI, body mass index; CXI, cachexia index; HR, hazard ratios; TNM stage, Tumor-node-metastasis stage; 95% CI, 95% confidence intervals.

## Data Availability

This published article included all the data of this study.
